# A comparative NMR-based metabolomics study of lung parenchyma of severe COVID-19 patients

**DOI:** 10.3389/fmolb.2023.1295216

**Published:** 2023-11-15

**Authors:** Joaquín I. Hurtado, Andrés López-Radcenco, José Luis Izquierdo-García, Fernando Rodríguez, Guillermo Moyna, Gonzalo Greif, Nicolás Nin

**Affiliations:** ^1^ Laboratorio de Interacción Hospedero Patógeno, Unidad de Biología Molecular, Institut Pasteur de Montevideo, Montevideo, Uruguay; ^2^ Departamento de Química del Litoral, Universidad de la República, Paysandú, Uruguay; ^3^ Grupo de Resonancia Magnética Nuclear e Imagen en Biomedicina, Instituto Pluridisciplinar, Universidad Complutense de Madrid, Madrid, Spain; ^4^ Departamento de Química en Ciencias Farmacéuticas, Facultad de Farmacia, Universidad Complutense de Madrid, Madrid, Spain; ^5^ Centro de Investigación Biomédica en Red de Enfermedades Respiratorias (CIBERES), Instituto de Salud Carlos III, Madrid, Spain; ^6^ Centro de Referencia COVID 1, Hospital Español, Administración de Servicios de Salud del Estado, Montevideo, Uruguay; ^7^ Centro de Referencia COVID 2, Instituto Nacional de Ortopedia y Traumatología, Administración de Servicios de Salud del Estado, Montevideo, Uruguay

**Keywords:** biomarkers, COVID-19, ICU patients, lung parenchyma, NMR-based metabolomics

## Abstract

COVID-19 was the most significant infectious-agent-related cause of death in the 2020-2021 period. On average, over 60% of those admitted to ICU facilities with this disease died across the globe. In severe cases, COVID-19 leads to respiratory and systemic compromise, including pneumonia-like symptoms, acute respiratory distress syndrome, and multiorgan failure. While the upper respiratory tract and lungs are the principal sites of infection and injury, most studies on the metabolic signatures in COVID-19 patients have been carried out on serum and plasma samples. In this report we attempt to characterize the metabolome of lung parenchyma extracts from fatal COVID-19 cases and compare them with that from other respiratory diseases. Our findings indicate that the metabolomic profiles from fatal COVID-19 and non-COVID-19 cases are markedly different, with the former being the result of increased lactate and amino acid metabolism, altered energy pathways, oxidative stress, and inflammatory response. Overall, these findings provide additional insights into the pathophysiology of COVID-19 that could lead to the development of targeted therapies for the treatment of severe cases of the disease, and further highlight the potential of metabolomic approaches in COVID-19 research.

## 1 Introduction

As experienced during the 2020–2023 COVID-19 pandemic, SARS-CoV-2 infections can result in a variety of respiratory conditions, including pneumonias-like symptoms, acute respiratory distress syndrome (ARDS), and multiorgan failure (Chavez et al., 2021). Potential risk factors for mortality among patients admitted to ICU included age, obesity, and comorbidities such as hypertension, diabetes, and cardiovascular disease (Ejaz et al., 2020; Ahlström et al., 2021; Booth et al., 2021). It was also observed that the clinical symptoms of COVID-19 could be influenced by viral load as well as by respiratory and gut microbiota dysbiosis (Liu et al., 2020; Brosseau et al., 2021). While most of the patients diagnosed with COVID-19 attended the disease at home, 13%-14% needed hospitalization in moderate care facilities, and between 5%-6% were admitted to intensive care units (Verity et al., 2020; Gosangi et al., 2022). Hospital mortality was between 30%–60% in case series reported in the first wave, increasing significantly for patients admitted to the ICU who required mechanical ventilation (Abate et al., 2020; Bastos et al., 2021; Estenssoro et al., 2021; Kurtz et al., 2021; Ranzani et al., 2021; Dongelmans et al., 2022). Uruguay was no exception, and towards the end of 2020 the average number of new cases increased exponentially to over 400 cases per day (GUIAD-COVID-19, 2022). In addition, the most prevalent viral variant during the first wave was B.1.1.28 (now designated as P.6), and vaccines were not yet available (Moreno et al., 2020; Elizondo et al., 2021; Rego et al., 2021).

A number of studies have established that SARS-CoV-2 infections set off a chain of events that can lead to a cytokine storm, an immune system overreaction that may result in ARDS (Koçak Tufan et al., 2021), which is the most frequent complication of severe COVID-19 cases. However, there are still several aspects of the disease that remain unknown. In order to elucidate the pathophysiological effects of COVID-19 and improve clinical care through the selection of appropriate treatments, particularly for patients with severe manifestations of the disease, a thorough understanding of the metabolic alterations and early acute lung injury biomarkers are required.

Metabolomic profiling can complement the lack of knowledge regarding the molecular mechanisms underlying clinical manifestations and pathogenesis of COVID-19. Consequently, several studies have employed metabolomic approaches to better understand the metabolic pathways involved in COVID-19 pathogenesis (Ansone et al., 2021; Chen et al., 2022; Murali et al., 2023). Serum-based metabolomic studies in COVID-19 patients revealed altered glycolytic pathways as well as amino acid, lipid, and anaplerotic metabolism, suggesting an impact on energy pathways, inflammatory response, and oxidative stress, and confirming the systemic nature of the disease (Kimhofer et al., 2020; Lorente et al., 2021; Shi et al., 2021; Valdés et al., 2022). Additionally, metabolomic studies have been conducted in different biofluids, including sweat, saliva and used face masks, as well as exhaled breath, serum and plasma, to identify differential metabolites and metabolic changes associated with COVID-19 (Barberis et al., 2020; Barberis et al., 2021; Hasan et al., 2021). However, there are no studies focusing on changes in the metabolic profile in lung tissue, which is SARS-CoV-2 primary site of infection. In the present communication we use an NMR-based non-targeted metabolomics approach to characterize the metabolome of lung parenchyma from fatal COVID-19 cases and compare it with other fatal respiratory diseases. As discussed herein, we found statistically significant differences between metabolites related to energy metabolism and inflammatory processes, revealing a unique metabolic profile in the infected tissue.

## 2 Materials and methods

### 2.1 Sample acquisition and experimental design

The inclusion criteria comprised adults 18 years or older admitted to the ICU with respiratory sepsis and respiratory failure and which had received mechanical ventilation. Clinical information was obtained by retrospective chart review, and data of the Acute Physiology and Chronic Health disease Classification System II (APACHE-II) scores on admission, arterial oxygen pressure/inspired fraction of oxygen (PaO_2_/FiO_2_ or PAFI), the need of vasopressor support, renal or multiorgan failure, and the presence of comorbidities, such as diabetes, hypertension, or obesity, were collected. Fragments of lung tissue were collected during clinical autopsies performed on ICU patients deceased between November 2020 and February 2021 who had SARS-CoV-2 infection confirmed by RT-qPCR (*n* = 8). As stated above there was no vaccination strategy in place at the time, and therefore none of these patients had received immunization. In addition, lung fragments from non-COVID-19 deceased patients were collected between December 2016 and June 2018 at the same facility and with the same ethical safeguards. This group included microbiological and serological positive results for *Klebsiella pneumoniae*, *Leptospira interrogans*, and respiratory syncytial virus (*n* = 7). In all cases, tissue samples were obtained in the first 2 h post-mortem and stored at −80 °C until processed for NMR analysis.

### 2.2 NMR sample preparation and data acquisition

An adaptation of previously published methods was followed (Nakayasu et al., 2016). Briefly, lung tissue samples between 50 and 100 mg in wet weight were homogenized and extracted with 0.7 mL MeOH/H_2_O (4:3) in a bullet blender (Next Advance, United States). Subsequently, chloroform was added to reach a final CHCl_3_/MeOH/H_2_O ratio of 8:4:3, vortexed for 5 min, and centrifuged for 5 min at 5,000 g. The aqueous phases were lyophilized and resuspended in a phosphate buffer prepared in D_2_O (pH 7.4) (Dona et al., 2014).

Water-suppressed 1D-NOESY ^1^H NMR spectra of aqueous tissue extracts were obtained at 25 °C on a Bruker AVANCE III 500 operating at a ^1^H frequency of 500.13 MHz. A spectral width of 10 kHz, a data size of 32 K, and a total of 128 scans were employed to record each spectrum, using a relaxation delay of 4 s between scans. 1D-TOCSY and HSQC spectra were acquired and processed using parameters provided with the spectrometer.

### 2.3 NMR data processing

NMR data were processed and analyzed with MNova (version 14.0, MestreLab Research, S.L., Santiago de Compostela, Spain). Free induction decays were zero-filled to 64 K points and apodized with a 0.3 Hz exponential window function prior to Fourier transformation. All spectra were manually phase- and baseline-corrected, and referenced to the anomeric proton signal of α-glucose (5.22 ppm). Spectra were manually aligned, and the data was normalized to the total spectral area after excluding the residual water resonance region and regions without signals. No binning was employed to construct the data matrices used for the multivariate statistical analyses.

### 2.4 Metabolite identification and estimation of relative concentrations

Metabolites were identified by comparison of ^1^H NMR data against spectral repositories, including the Biological Magnetic Resonance Bank (BMRB) (Hoch et al., 2023), the Human Metabolome Database (HMDB) (Wishart et al., 2022), and Chenomx (version 9, Chenomx, Inc., Edmonton, Canada). When required, metabolite identification was confirmed with data from 1D-TOCSY and HSQC spectra.

Given the characteristics of lung parenchyma and the difficulties of obtaining precise dry weights in biologically-hazardous samples, variations in metabolite levels were estimated using relative concentrations. This figure was computed as the ratio between the area from individual metabolite ^1^H NMR signals and the total area of the spectrum.

### 2.5 Statistical analysis

Multivariate statistical analyses, including principal component analysis (PCA) and orthogonal partial least squares discriminant analysis (OPLS-DA), were carried out with the PLS_Toolbox package (version 8.5, Eigenvector Research Inc., Manson, WA, United States) implemented for MATLAB (revision 2014a, The MathWorks Inc., Natick, MA, United States). For all models, the data was mean-centered and scaled using a Pareto factor (Van Den Berg et al., 2006). Analysis of the data was first performed with PCA, which reduces data dimensionality and facilitates the identification of clusters or trends (Wold et al., 1987; Trygg and Wold, 2002; Trygg et al., 2006). The PCA scores plot was also employed to identify strong outliers outside the 95% significance region of Hotelling’s T2 ellipse. Cross-validation of OPLS-DA models was achieved using the random subset method, which involved 20 iterations over data split into 5 equally-sized parts. Receiver operating characteristic (ROC) curves were plotted, and areas under the curves were calculated to ensure the goodness of fit of the resulting models (Ekelund, 2012; Simundic, 2012). Permutation tests with 100 iterations were also performed to determine the degree of over-fitting and further validate the discriminant analyses (Ni et al., 2008). When needed, statistical total correlation spectroscopy (STOCSY) analyses were performed with an in-house MATLAB script based on the algorithm described elsewhere (Cloarec et al., 2005).

Pairwise *t*-test comparisons were carried out between continuous demographic variables as well as between the relative concentrations of all identified metabolites in COVID-19 and non-COVID-19 samples using GraphPad Prism (version 7.0, GraphPad Software, Inc., San Diego, CA, United States).

### 2.6 Metabolic pathways analyses

Metabolic pathway analysis was performed using the Pathway Analysis module of Metaboanalyst v.5.0 (Xia et al., 2011; Chong et al., 2019), which combines results from robust pathway enrichment analysis with pathway topology analysis to identify the most relevant pathways involved in the conditions under study (Aittokallio and Schwikowski, 2006; Kankainen et al., 2011). The selected pathway enrichment analysis method was GlobalAncova (Hummel et al., 2008), the node importance measure for topological analysis was out-degree centrality, and KEGG metabolic pathways were used as the backend knowledgebase.

## 3 Results

### 3.1 Clinical characteristics of study patients

All patients in this study had been diagnosed with pneumonia, presented respiratory sepsis, and exhibited high APACHE-II scores upon admission to the ICU (Table 1). They all required mechanical ventilation, and more than 80% were on vasopressor support. The average ICU stay was 17.6 ± 4.9 days for COVID-19, and 11.2 ± 8.3 for non-COVID-19 patients. When compared to non-COVID patients, those with COVID-19 had a higher percentage of comorbidities on admission (diabetes, hypertension, chronic obstructive pulmonary disease, and obesity) and a lower PAFI score.

**TABLE 1:** Demographic and clinical characteristics of the study population upon admission in ICU. Variations in continuous variabbles with *p*-values <0.05 are indicated with bold numbers.

Parameter	COVID-19	Non-COVID-19	*p*-value
Cohort size (*n*)	8	7	-
Mean age	68.6 ± 8.2	57.3 ± 17.1	0.992
Female	3 (37%)	3 (50%)	-
COPD^a^	4 (50%)	2 (33%)	-
Diabetes	3 (38%)	0 (0%)	-
Hypertension	7 (88%)	1 (17%)	-
Obesity	3 (38%)	0 (0%)	-
Renal failure	5 (63%)	2 (33%)	-
APACHE-II score	20.6 ± 8.4	19.2 ± 10.2	>0.999
PAFI^b^ on day 1	115 ± 31	230 ± 162	**0.001**
Vasopressor support	8 (100%)	5 (83%)	-
Days of mechanical ventilation	16.4 ± 5.3	9.7 ± 7.1	>0.999
Length of ICU stay	17.6 ± 4.9	11.2 ± 8.3	>0.999

^a^ Chronic obstructive pulmonary disease.

^b^ Arterial oxygen pressure/inspired fraction of oxygen (PaO2/FiO2 or PAFI).

### 3.2 Metabolomic analysis

**FIGURE 1:**
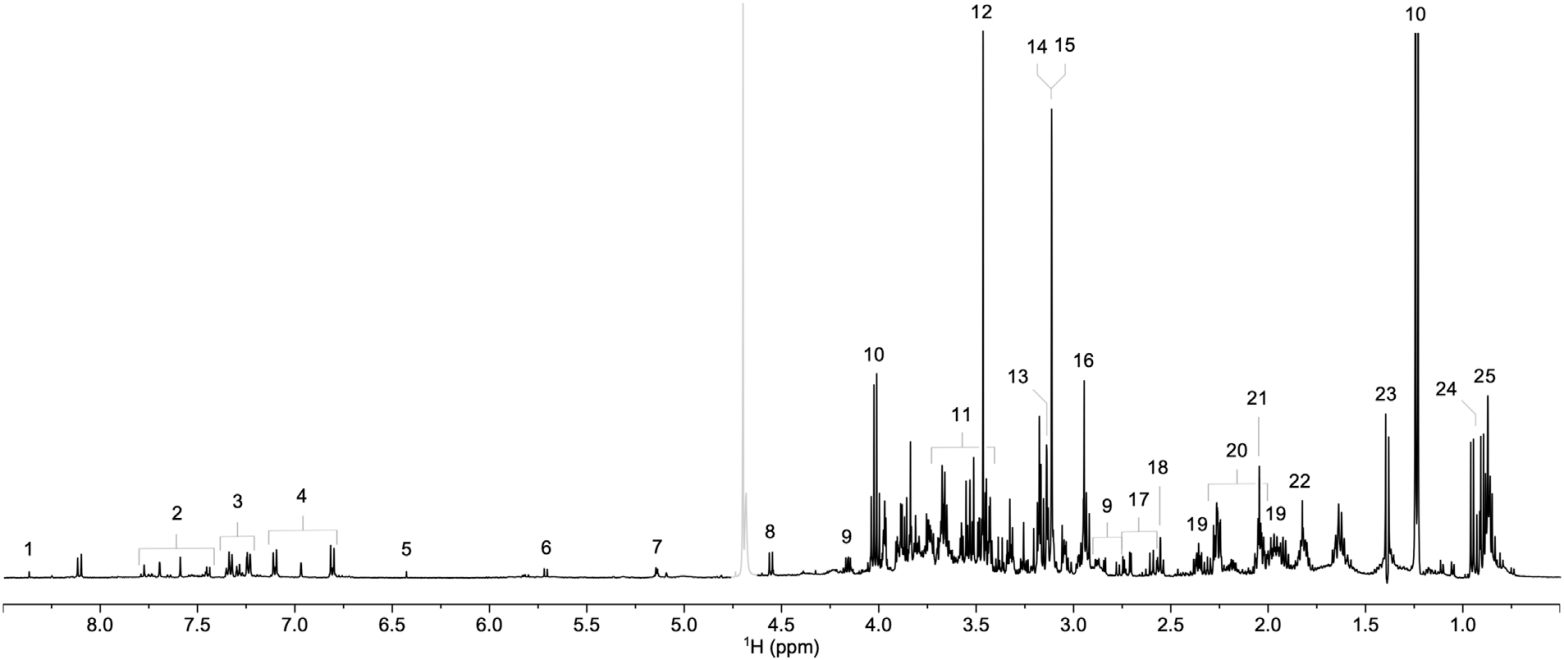
Representative ^1^H NMR spectrum of a lung parenchyma extract sample. Signals corresponding to formate (1), tryptophan (2), phenylalanine (3), tyrosine (4), fumarate (5), uracil (6), α-glucose (7), β-glucose (8), asparagine (9), lactate (10), glycerol-3-phosphate (11), glycine (12), betaine (13), choline (14), phosphocholine (15), creatine (16), citrate (17), pyruvate (18), glutamine (19), glutamate (20), methionine (21), acetate (22), alanine (23), valine (24), and isoleucine (25) are annotated. The grayed-out region corresponds to the residual HDO signal.

**FIGURE 2:**
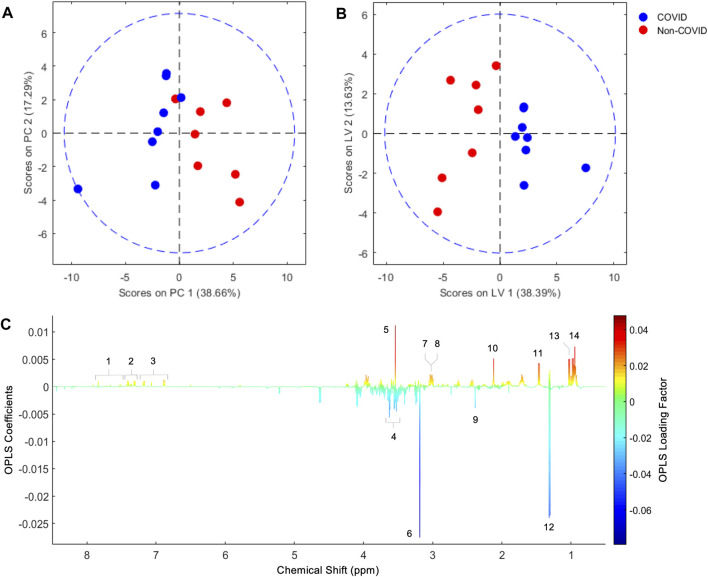
PCA score plot obtained from lung parenchyma extract ^1^H NMR data **(A)**, and score and loading factor plots obtained from the OPLS-DA comparing COVID-19 and non-COVID samples (**B** and **C**, respectively). Metabolites that differentiate the COVID-19 from the non-COVID-19 cohorts are annotated in the loading factor plot, including tryptophan (1), phenylalanine (2), tyrosine (3), glycerol-3-phosphate (4), glycine (5), choline (6), creatine (7), asparagine (8), succinate (9), methionine (10), alanine (11), lactate (12), valine (13), and isoleucine (14). The OLPS-DA model had R^2^Y and Q^2^Y coefficients of 0.75 and 0.32, respectively, and its ROC curve had an AUC value of 0.98 (Supplementary Figures S1, S2).

We initially compared ^1^H NMR profiles from lung tissue extracts from COVID-19 autopsies against those from non-COVID-19 autopsies (Figure 1). As shown in Figure 2A, a PCA derived from the ^1^H NMR data showed good discrimination between groups despite the low number of samples. Indeed, inspection of the loading plot from an OPLS-DA model obtained with the same data identified an important number of discriminating ^1^H signals (Figures 2B, C). Dereplication using a combination of STOCSY analyses, classical 1D and 2D NMR experiments, and comparison to data from various ^1^H spectral repositories allowed us to identify 21 metabolites (Figure 2C), 11 of which had significant differences in levels among the two cohorts (Table 2). The relative concentrations of the amino acids valine, alanine, methionine, glycine, tryptophane, phenylalanine, tyrosine, and asparagine were significantly increased in samples from COVID-19 patients. On the other hand, choline and glycerol-3-phosphate levels, as well as that of the metabolic intermediate succinate, were significantly lower among these samples.

**TABLE 2:** Metabolite relative concentrations in COVID-19 and non-COVID-19 patients. Variations with *p*-values <0.05 are indicated with bold numbers.

Metabolite	COVID-19	Non-COVID-19	Fold change^a^	*p*-value
Alanine	1.842 ± 0.205	1.306 ± 0.304	−1.41	**0.001**
Asparagine	0.145 ± 0.047	0.070 ± 0.030	−2.07	**0.002**
β-Hydroxybutyrate	0.246 ± 0.116	0.205 ± 0.063	−1.20	0.217
Betaine	0.395 ± 0.330	0.330 ± 0.211	−1.20	0.327
Choline	3.262 ± 0.808	5.341 ± 1.662	1.64	**0.008**
Creatine	0.655 ± 0.236	0.523 ± 0.214	−1.25	0.139
Glucose	0.269 ± 0.090	0.679 ± 0.561	2.52	0.081
Glutamate	2.309 ± 0.426	2.465 ± 0.488	1.07	0.263
Glycine	1.645 ± 0.229	1.202 ± 0.207	−1.37	**0.001**
Glycerol-3-phosphate	0.027 ± 0.004	0.036 ± 0.006	1.33	**0.002**
Histidine	0.129 ± 0.123	0.040 ± 0.014	−3.25	0.052
Isoleucine	1.508 ± 2.527	0.400 ± 0.132	−3.77	0.128
Lactate	15.958 ± 4.933	17.677 ± 3.053	1.11	0.214
Methionine	0.212 ± 0.116	0.109 ± 0.038	−1.94	**0.021**
Phenylalanine	0.564 ± 0.254	0.272 ± 0.067	−2.07	**0.007**
Phosphocholine	1.050 ± 0.342	1.010 ± 0.250	−1.04	0.400
Succinate	0.006 ± 0.002	0.013 ± 0.004	2.17	**0.002**
Tyrosine	0.415 ± 0.134	0.180 ± 0.052	−2.31	**0.001**
Tryptophan	0.054 ± 0.026	0.028 ± 0.003	−1.92	**0.011**
Uracil	0.074 ± 0.018	0.059 ± 0.022	−1.25	0.102
Valine	1.480 ± 0.677	0.723 ± 0.246	−2.05	**0.008**

^a^ Fold changes were computed according to the guidelines of Vinaixa and coworkers (Vinaixa et al., 2012).

### 3.3 Pathway analysis results

**FIGURE 3:**
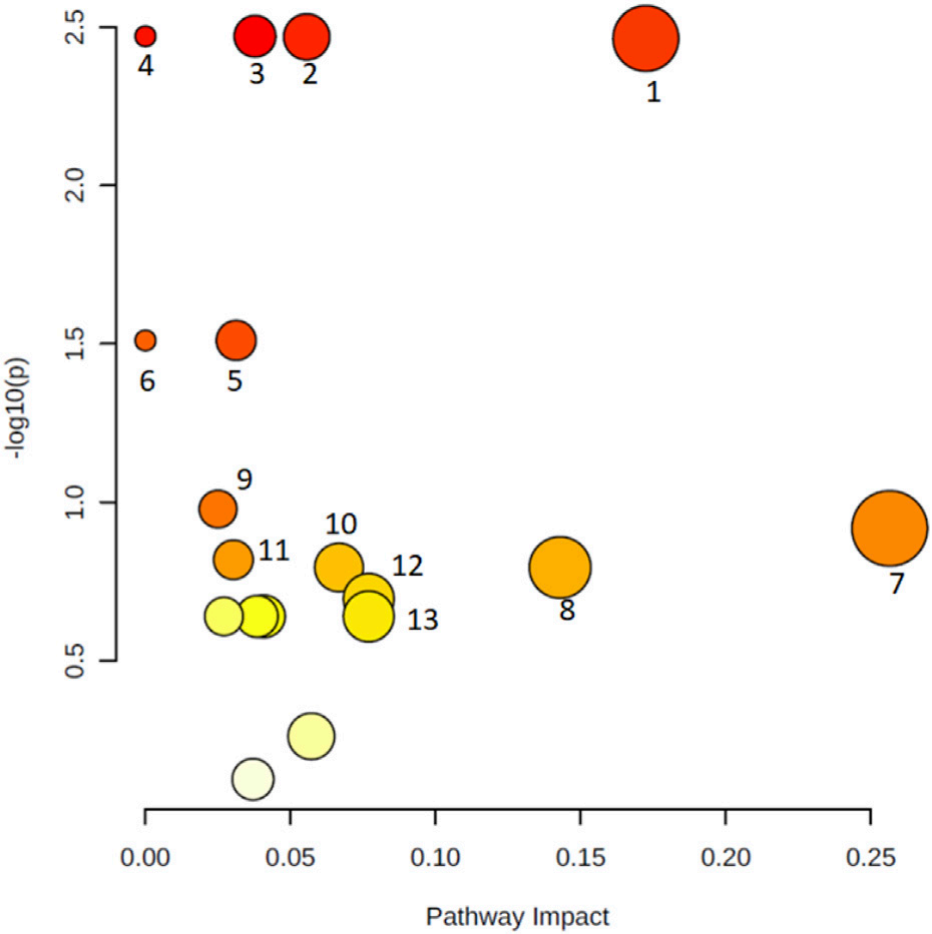
Metabolic pathway analysis of the set of metabolites identified in lung autopsies of COVID-19 patients and other fatal pneumonias. Y-axis represents the statistical *p*-values from enrichment analysis, and the X-axis represents the pathway impact value calculated from pathway topology analysis. The node colors represent the *p*-values (lowest: light yellow; highest: dark red) and the node radius indicate the pathway impact values. Dysregulated metabolic pathways include aminoacyl-tRNA biosynthesis (1), pantothenate and CoA biosynthesis (2), valine, leucine, and isoleucine degradation (3), valine, leucine, and isoleucine biosynthesis (4), alanine, aspartate, and glutamate metabolism (5), selenocompound metabolism (6), glycine, serine, and threonine metabolism (7), synthesis and degradation of ketone bodies (8), arginine and proline metabolism (9), butanoate metabolism (10), Cysteine and methionine metabolism (11), Glycerophospholipid metabolism (12), and glyoxylate and dicarboxylate metabolism (13).

Metabolic pathway analysis was performed to identify the most relevant pathways involved in COVID-19 lung autopsy (Figure 3). This pathway analysis identified alterations in amino acids biosynthesis and degradation, anaplerotic alanine-aspartate-glutamate metabolism, glycine-serine-threonine metabolism, synthesis and degradation of ketone bodies and glycerophospholipid metabolism.

## 4 Discussion

One of the most salient aspects from the results presented above is the general increase in the levels of essential amino acids, generally recognized as sepsis biomarkers (Mierzchala-Pasierb et al., 2020; Ahn et al., 2021), in patients with COVID-19. Indeed, branched chain amino acids (BCAAs), including isoleucine and valine (Table 2), are involved in stress, energy, and muscle metabolism (Neinast et al., 2019). BCAAs have different metabolic routes, with valine going solely to carbohydrates (glucogenic), leucine solely to fats (ketogenic), and isoleucine being both a glucogenic and a ketogenic amino acid. These metabolites can also regulate immune responses and influence viral infection (Atila et al., 2021). Hence, the maintenance of metabolic homeostasis is essential for the body’s normal physiological functioning, and disruptions in metabolic homeostasis could potentially facilitate virus infection. Our results in lung autopsies of COVID-19 patients show a significant enrichment in valine (Table 2). This is also evidenced by the metabolic pathway analysis, which revealed that valine, leucine, and isoleucine degradation and, to a lesser extent synthesis pathways, are significantly affected (Figure 3). High levels of BCAAs are associated with metabolic encephalopathy, often linked with respiratory suppression, epileptic seizures, and brain damage due to lack of oxygen (Ozturk et al., 2022). These results contrast those from a previous study conducted in serum, where the metabolic profiles of patients with ARDS due to COVID-19 and H1N1 were compared (Lorente et al., 2021). This report by Lorente and coworkers is particularly noteworthy, as it presents a footprint analysis in patients with the same severity of ARDS. On the other hand, most existing metabolomic studies contrast SARS-CoV-2 infected patients with healthy controls and cannot discern between metabolic dysregulations caused by the virus or the development of ARDS. These authors found that amino acid metabolism was decreased in COVID-19 patients, and the concentration of BCAAs, including isoleucine and valine, were also lower when compared with influenza A patients. Although different biofluids are commonly used for biomarker discovery, it is necessary to consider lung tissue metabolome as a complementary input. Indeed, it is not uncommon to find that certain metabolites are decreased in serum but increased in the tissue (Bernatchez and McCall, 2020).

Other metabolites found to be significantly more abundant in patients with COVID-19 were tyrosine, phenylalanine, and tryptophan. Absorption of the latter metabolite is mediated by angiotensin converting enzyme 2 (ACE2), the primary receptor of SARS-CoV-2, and has been recognized as a marker of inflammation in severe COVID-19 cases (Takeshita and Yamamoto, 2022). Similarly, elevated plasma or serum levels of tyrosine are observed in a variety of ailments, including hyperphenylalaninemia, sepsis, severe burns, transient tyrosinemia and hyperphenylalaninemia of the newborn, phlebotomus fever, viral hepatitis, or hepatic encephalopathy (Rosen et al., 1977; Watanabe et al., 1979; Rudnick and Ebach, 2004; Ansone et al., 2021). High levels of this non-essential amino acid synthetized from phenylalanine have also been detected in septic patients (Freund et al., 1978). Also, increased phenylalanine serum concentrations have been associated with immunological activation and an increased risk of cardiovascular events in sepsis and other viral infections (Ansone et al., 2021). This could be explained due to muscle tissue catabolism leading to amino acid release, which, together with the body’s differential metabolic capacity for different amino acids, results in their accumulation. Indeed, despite muscle tissue is easily able to oxidize BCAAs to support its own energy requirements, aromatic amino acids as well as sulfur-containing amino acids such as taurine, cysteine, and methionine are not as easily metabolized, and may account for the increase in the levels of tyrosine seen during sepsis (Freund et al., 1978). It has also been reported that as disease severity progresses, there is a significant increase in phenylalanine serum concentrations (Martínez-Gómez et al., 2022). Taken together with our results, these findings support the idea that these aromatic amino acids could be used as biomarkers of COVID-19 severity.

Additionally, succinate was found significantly depleted in COVID-19 patients. This metabolite plays a key role in hypoxia, where it acts inhibiting the prolyl hydroxylase domain-containing enzymes (PHD) (Yang et al., 2012). Under normal oxygenation, PHD constantly degrades the hypoxia-inducible transcription factor (HIF). This O_2_-sensitive factor mediates the response to hypoxia through the expression of genes that regulate cellular energy production, biosynthesis, cell growth, and redox homeostasis (Yang et al., 2014). In our cohort of severe COVID-19 patients lower initial PAFI scores were observed, indicating decreased blood oxygenation (Yang et al., 2012). While increased succinate levels would be expected in this scenario, it is known that mechanical ventilation periods like the ones experienced by our patients lead to succinate downregulation (Mussap and Fanos, 2021). As previously reported, these results indicate that despite high sensitivity, changes in succinate levels are not suitable indicators of disease severity or patient prognosis (Mussap and Fanos, 2021).

Choline levels were also found to be significantly lower in COVID-19 samples. This has also been reported in serum from severe COVID-19 patients, where an increase in the consumption of this trimethylamine caused by activation of macrophage innate immune receptors was linked to extracellular cytokine secretion (Sanchez-Lopez et al., 2019). The presence of pro-inflammatory components in bronchoalveolar lavage fluid is elevated even in severe COVID-19 patients treated with glucocorticoids, suggesting that slowing down the cytokine storm is a critical strategy for disease control (Barberis et al., 2020).

Similarly, we found a significant drop in glycerol-3-phosphate levels among COVID-19 samples. This phosphorylated polyol is tightly related to phospholipid metabolism, which is now known to be deregulated in COVID-19 patients based on serum metabolomic analyses (Shen et al., 2020; Shi et al., 2021). More importantly, it has been reported that the decrease in the levels of this species are directly related to severity in COVID-19 patients (Wu et al., 2020). Although the reduction in glycerol-3-phosphate concentration at the site of SARS-CoV-2 infection warrants further investigation, our results corroborate that this metabolite could be considered as a biomarker of severe manifestations of the disease.

Finally, lactate was the most widely expressed metabolite across both cohorts with no statistically significant differences between them. This finding is consistent with the known fact that high plasma lactate concentration is a marker of poor prognosis and an indicative of metabolic acidosis in critically ill patients, and was expected to be higher in both groups (Martha et al., 2021).

In conclusion, distinct metabolic signatures associated with energy metabolism and inflammatory pathways differentiate COVID-19 from fatal pneumonias caused by other respiratory infections. In particular, we found a significant increase in the levels of branched-chain, aromatic, and sulfur-containing amino acids in lung tissue from fatal COVID-19 cases. Many of these have been recognized as sepsis and inflammatory markers and are associated with lung injury, a condition that commonly leads to severe refractory hypoxemia and is one of the main causes of mortality in COVID-19 patients (Dhont et al., 2020; Donina, 2022; Ribeiro et al., 2022).

To our knowledge, this is the first comparative metabolomic study employing lung tissue samples from COVID-19 patients. In spite of the heterogeneity and wide range of symptoms observed, our findings provide additional insights into the pathogenesis of COVID-19 and have helped identify potential biomarkers for disease severity and treatment efficacy. Notwithstanding, the nature of the samples led to small cohorts affected differently by comorbidities. Some of these, such as diabetes, could have a sizable impact on the metabolic pathways identified as altered in our analyses (Felig et al., 1977). Therefore, the preliminary results reported in this work should be further corroborated in larger scale studies.

## Data Availability

The dataset presented in this study can be found at Mendeley Data with doi: 10.17632/bg45mx8rxd.1 (https://data.mendeley.com/datasets/bg45mx8rxd).
