# Supplementary material for: A comparative NMR-based metabolomics study of lung parenchyma of severe COVID-19 patients
Source: Front Mol Biosci. 2023 Nov 15;10:1295216. doi: 10.3389/fmolb.2023.1295216 (PMC10684917; doi:10.3389/fmolb.2023.1295216)
Supplement: Supplementary file 1 [file Table1.DOCX]

Supplementary Material


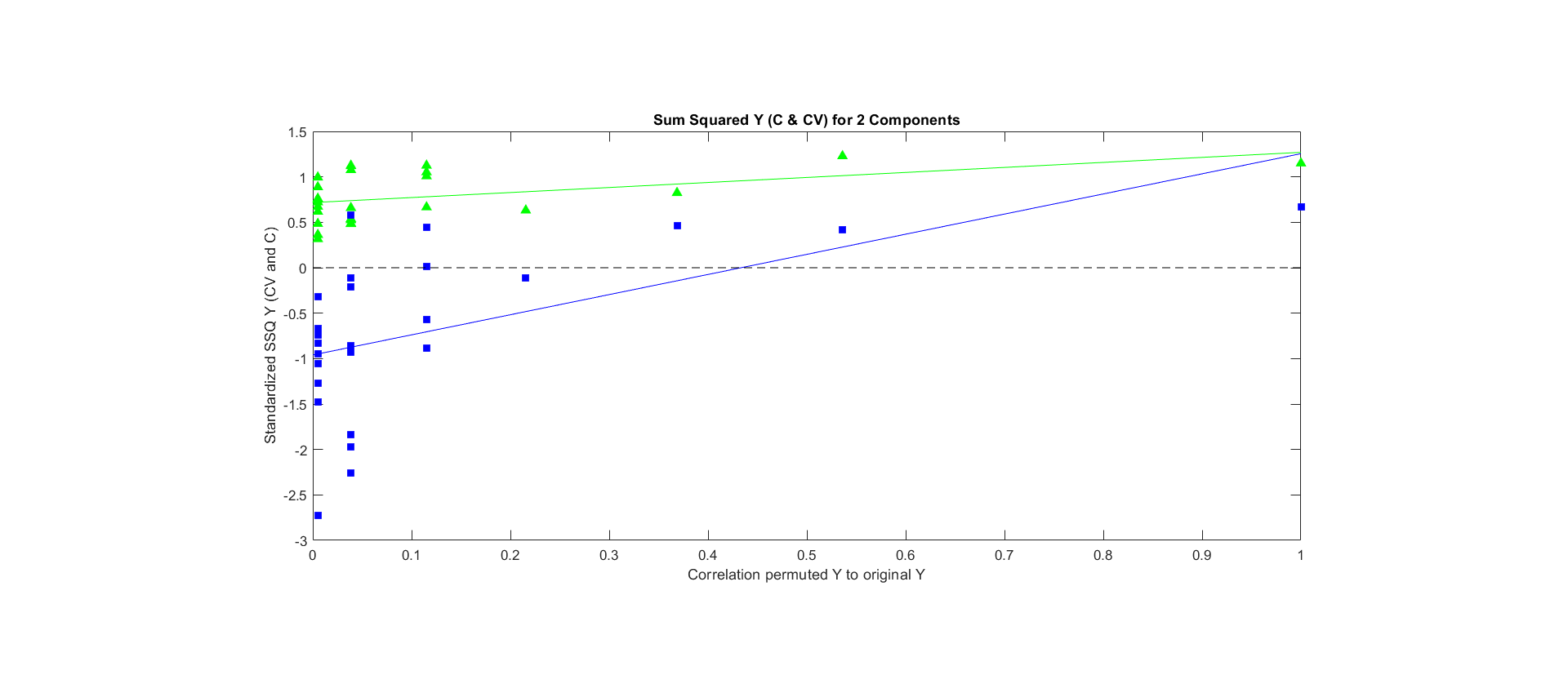


**Figure S1**. Permutation test plots for the OPLS-DA model comparing tissue extracts from COVID‑19 autopsies against those from non-COVID-19 autopsies (R^2^Y = 0.75 and Q^2^Y = 0.32).


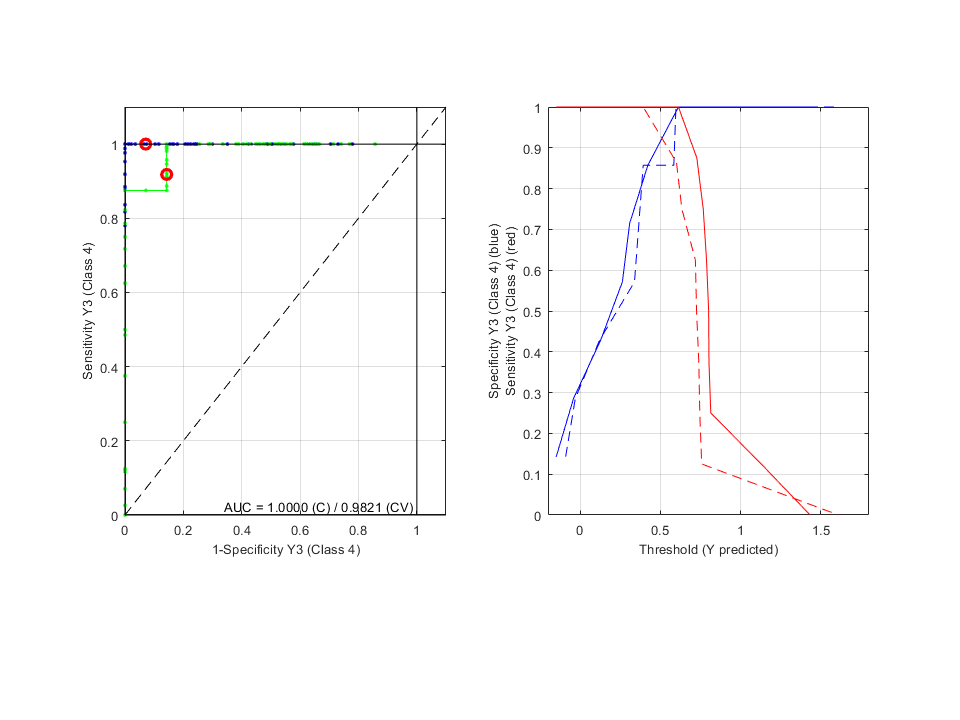


Figure S2. ROC analysis curves derived from the OPLS-DA model comparing tissue extracts from COVID-19 autopsies against those from non-COVID-19 autopsies (AUC = 0.98).
